# Organ‐Dependent Antioxidant, Redox‐Modulating, and Lifespan Effects of *Galanthus elwesii* Extracts in *Drosophila melanogaster*

**DOI:** 10.1002/fsn3.72055

**Published:** 2026-06-25

**Authors:** Osman Ertan, Arif Ayar, Ebru Batı Ay

**Affiliations:** ^1^ Department of Biotechnology, Institute of Science Amasya University Amasya Türkiye; ^2^ Department of Medical Services and Techniques, Sabuncuoğlu Şerefeddin Health Services Vocational School Amasya University Amasya Türkiye; ^3^ Department of Plant and Animal Production, Suluova Vocational School Amasya University Amasya Türkiye

**Keywords:** Drosophila aging, *Galanthus elwesii*, lifespan modulation, oxidative stress biomarkers, redox homeostasis

## Abstract

*Galanthus elwesii* is a pharmaceutically valuable Amaryllidaceae species known for its diverse bioactive metabolites; however, the relationship between its phytochemical profile, antioxidant capacity, and in vivo biological effects remains poorly understood. In this study, aerial (APE) and belowground (BPE) parts were extracted using water, methanol, and ethanol, and evaluated through a combined in vitro and in vivo approach. Total phenolic and flavonoid contents were quantified, and antioxidant activities were assessed via DPPH radical‐scavenging and Fe^2+^ metal‐chelation assays, including IC_50_ determination. The in vivo effects of selected extracts were investigated in *Drosophila melanogaster* through larval toxicity and developmental survival assays, adult lifespan analysis, and oxidative stress biomarkers (TAS, TOS, and OSI). Phytochemical composition and antioxidant activity exhibited strong organ‐ and solvent‐dependent variability, with belowground extracts generally demonstrating higher phenolic content and stronger radical‐scavenging and metal‐chelating capacities. In vivo, most extracts showed low‐to‐moderate developmental toxicity at selected concentrations, while certain belowground extracts significantly extended lifespan at subtoxic doses. Oxidative status measurements revealed reduced TOS and OSI levels alongside maintained or increased TAS, indicating effective suppression of oxidative stress rather than direct antioxidant action. Correlation analysis revealed moderate negative correlations between phenolic/flavonoid content and antioxidant activity, although these relationships did not reach statistical significance. Overall, these findings suggest that *G. elwesii* extracts exert organ‐dependent phytochemical and bioactivity profiles and may act as in vivo redox modulators associated with improvements in oxidative balance and lifespan‐related parameters.

## Graphical abstract

Aerial (leaves and flowers) and belowground (bulbs and roots) parts of *Galanthus elwesii* were extracted using water, 70% ethanol, and 80% methanol. Phytochemical analyses revealed organ‐ and solvent‐dependent variations in total phenolic and flavonoid contents, which were associated with differences in in vitro antioxidant activity determined by DPPH radical‐scavenging and Fe^2+^ metal‐chelating assays. In vivo evaluation using *Drosophila melanogaster* demonstrated improved oxidative balance through increased total antioxidant status (TAS) and reduced total oxidant status (TOS) and oxidative stress index (OSI). Selected extracts also extended lifespan, indicating the antioxidant and potential health‐promoting properties of *G. elwesii* extracts.

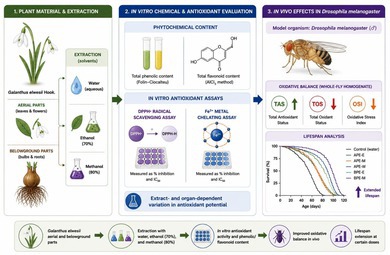

## 1 Introduction

Oxidative stress plays a fundamental role in the pathogenesis of many chronic diseases, including cardiovascular diseases, diabetes, neurodegenerative disorders, and cancer (Pizzino et al. 2017; Sharifi‐Rad et al. 2020). Disruption of antioxidant defense mechanisms, including key enzymes such as paraoxonase‐1 (PON1) that protect lipoproteins against oxidative modification, has been closely linked to the onset and progression of these pathological conditions (Demir 2020). Imbalance between free radical production and antioxidant defense systems may contribute to cellular damage accumulation and age‐related functional decline (Finkel and Holbrook 2000; Luo et al. 2020). Therefore, the identification of natural compounds and plant‐derived products capable of reducing oxidative stress and enhancing antioxidant capacity has gained increasing importance in both contemporary and traditional medical practices (Pisoschi and Pop 2015; Scuto et al. 2022; Sharma and Sharma 2024). Plant‐derived phenolic compounds, owing to their structural diversity and multiple redox‐modulating mechanisms, have attracted considerable scientific interest as potential contributors to oxidative balance regulation (Riaz et al. 2023; Sağlamtaş et al. 2025).

Türkiye is recognized as one of the world's major plant gene centers due to its diverse climate, topography, and varied ecological niches. The country harbors a high level of endemic plant diversity, with geophyte plants possessing bulbs, tubers, or rhizomes constituting a significant component of its flora (Aydın and Mammadov 2022; Fırat et al. 2015; Özhatay et al. 2013). Geophytes are in high demand not only because of their bioactive phytochemical constituents but also due to their economic value as ornamental plants (Ay et al. 2023).

The genus *Galanthus*, belonging to the Amaryllidaceae family, is particularly notable for its medically important alkaloids. Especially *Galanthus elwesii* (snowdrop) exhibits anticholinesterase, neuroprotective, anti‐inflammatory, antiviral, antimicrobial, and antioxidant properties due to its Amaryllidaceae alkaloids, such as galantamine (Heinrich and Teoh 2004; Berkov et al. 2011; Kaya et al. 2014; Nair and van Staden 2024). Galantamine is a well‐established acetylcholinesterase inhibitor used in the symptomatic treatment of Alzheimer's disease, highlighting the pharmacological potential of Amaryllidaceae alkaloids (Lilienfeld 2002; Cusi et al. 2007; He et al. 2015). Recent reviews and experimental studies have revealed that alkaloids and polyphenols derived from *Galanthus* species possess a broad spectrum of biological activities, including antioxidant, anticancer, and antiaging effects (Benedec et al. 2018; Georgiev et al. 2024). In particular, *G. elwesii* has been reported to exhibit high phenolic and flavonoid content as well as strong in vitro antioxidant activity, suggesting that it could serve as a promising natural antioxidant source (Ay et al. 2020).

The role of the antioxidant defense system in aging biology is considered one of the major mechanisms associated with lifespan regulation. According to the free radical/oxidative stress theory of aging, excessive production of reactive oxygen species (ROS) and insufficient detoxification lead to irreversible damage to cellular macromolecules such as DNA, lipids, and proteins, thereby accelerating the aging process (Liguori et al. 2018; Yang et al. 2024). Experimental evidence from in vivo models further demonstrates that ROS‐mediated macromolecular damage disrupts key transcription factors and mitochondrial regulatory networks, potentially contributing to apoptotic signaling and organ‐specific toxicity (Demir et al. 2025; Özturk et al. 2024). The balance between antioxidant enzymes (e.g., SOD, CAT, GPx, GR) and nonenzymatic antioxidants (e.g., glutathione, bilirubin, albumin, vitamins C and E, coenzyme Q10) determines an organism's resistance to oxidative stress. The coordinated activity of these antioxidant defense components is essential for maintaining cellular redox balance under both physiological and stress conditions (Pisoschi and Pop 2015; Ighodaro and Akinloye 2018; Sies and Jones 2020; Özdemir and Demir 2025). Accordingly, in vivo evaluation of plant‐derived antioxidants may provide valuable insight into their physiological relevance in aging and oxidative stress biology (Sharma and Sharma 2024).

The use of model organisms in biomedical research has greatly facilitated the elucidation of molecular mechanisms. Among these, *Drosophila melanogaster* (fruit fly) stands out due to its genetic tractability, short life cycle, low maintenance cost, ease of culture, and absence of ethical constraints (Hales et al. 2015; Mirzoyan et al. 2019; Victor Atoki et al. 2025). The *D. melanogaster* genome is highly conserved with that of humans; approximately 60%–70% of human disease‐related genes have homologs in the fly, making it an effective model for studying the molecular basis of many human diseases (Reiter et al. 2001; Ugur et al. 2016; Obafemi et al. 2025). In particular, *D. melanogaster* is widely used in aging, neurodegeneration, and oxidative stress research due to its genetic manipulability and relatively short lifespan (Akinade et al. 2022; Fidan and Ayar 2022; Casas‐Tintó 2024; Zhang et al. 2024; Afolayan et al. 2025; Buacheen et al. 2025).

Although numerous studies have investigated the chemical composition and in vitro antioxidant activities of *Galanthus* species, research evaluating the in vivo biological effects of *G. elwesii* extracts in experimental aging models remains limited (Ay et al. 2020, 2023). In particular, comparative investigations examining whether extracts obtained from different plant organs produce distinct physiological responses under in vivo conditions are lacking. Furthermore, although in vitro antioxidant assays provide important preliminary information regarding extract bioactivity, their physiological relevance cannot always be directly extrapolated to whole‐organism systems (Sharma and Sharma 2024). Therefore, integrative experimental approaches combining in vitro antioxidant evaluation with in vivo biological models are important for better understanding the potential redox‐associated effects of medicinal plant extracts.

In the present study, six extracts obtained from the aboveground (APE) and belowground (BPE) parts of *G. elwesii* using solvents of varying polarity (water, methanol, and ethanol) were investigated in a two‐phase experimental design. Initially, total phenolic and flavonoid contents together with in vitro antioxidant activities (DPPH radical‐scavenging and metal‐chelating activity) were determined to comparatively characterize the extracts. In the second phase, the biological effects of selected extracts were evaluated in *Drosophila melanogaster* , with particular emphasis on developmental toxicity, lifespan‐associated responses, and systemic oxidative stress biomarkers, including TAS, TOS, and OSI. We hypothesized that *G. elwesii* extracts may modulate oxidative balance and lifespan‐related parameters in *D. melanogaster* under subtoxic exposure conditions, and that these responses could vary depending on the plant organ and extraction solvent.

## 2 Materials and Methods

### 2.1 Plant Material and Extraction Procedure

In this study, *G. elwesii* Hook. (Taurus snowdrop) bulbs with a diameter > 4 cm were obtained from a commercial supplier. The bulbs were planted in pots at the end of September and harvested at the end of March. Collected plant samples were washed with tap water to remove potential environmental contaminants. The cleaned bulbs were then placed on drying paper in a clean environment and left to dry for 2 weeks. After drying, samples were labeled and stored in appropriate containers until further processing.

The underground (bulb and roots) and aboveground (flowers and fruits) parts of *G. elwesii* were analyzed separately. A total of six different extracts were prepared from these parts using water, methanol, and ethanol as solvents. Dried plant materials were ground into powder using a mill and stored in 50 mL Falcon tubes at room temperature.

For pre‐extraction, 5 g of powdered plant material was weighed using an analytical balance and transferred into capped glass containers. Subsequently, 200 mL of methanol, ethanol, or distilled water was added to each sample. The mixtures were subjected to maceration at room temperature for 72 h using a shaker. At the end of this period, plant suspensions were filtered through filter paper to separate the liquid phase from the plant residue. The filtrates were then concentrated by removing solvents using a rotary evaporator.

### 2.2 Determination of Total Flavonoid Content

Total flavonoid content was determined following the method described by Park et al. (2008), using quercetin as a standard. For the analysis, 1 mL of plant extract was transferred into test tubes, followed by the addition of 2 mL distilled water, 0.15 mL of 0.5 M NaNO_2_, and 0.15 mL of 0.3 M AlCl_3_ solution. After 5 min, 1 mL of NaOH was added to terminate the reaction. The absorbance of the resulting colored complex was measured at 510 nm using a spectrophotometer.

Flavonoid content was calculated by comparing absorbance values with a quercetin standard calibration curve. Results were expressed as milligrams of quercetin equivalent per gram of dry extract (mg QE/g).

### 2.3 Determination of Total Phenolic Content (Folin–Ciocalteu Method)

Total phenolic content was determined according to the method of Slinkard and Singleton (1977) using Folin–Ciocalteu reagent and gallic acid as standard. For the assay, 1 mL of plant extract was mixed with 4.5 mL distilled water and 0.1 mL Folin–Ciocalteu reagent, then incubated in the dark for 5 min. Subsequently, 0.3 mL of 2% sodium carbonate solution was added.

The tubes were sealed with parafilm and incubated in the dark for 1 h. Absorbance was then measured at 765 nm using a spectrophotometer. Total phenolic content was calculated based on a gallic acid standard curve and expressed as milligrams of gallic acid equivalent per gram of dry extract (mg GAE/g).

### 2.4 DPPH Free Radical‐Scavenging Activity

Antioxidant activity was evaluated using the DPPH radical‐scavenging assay (Gopalraaj and Velayudhannair 2024; Açıkgöz et al. 2025). Plant extracts were prepared at concentrations ranging from 25 to 400 μg/mL. A 0.5 mL aliquot of each extract was mixed with 3 mL ethanol and 300 μL of a 20 mg/L DPPH solution. Butylated hydroxyanisole (BHA), butylated hydroxytoluene (BHT), and Trolox (100–500 μg/mL) were used as positive controls. The mixtures were shaken thoroughly, and absorbance was measured at 517 nm.

For the control group, 0.75 mL distilled water was used instead of extract. Radical‐scavenging activity (%) was calculated using the following formula:

$$\text{Radical}‐\text{Scavenging Activity}\;\left(\%\right)=\left(\frac{A_{0}-A_{1}}{A_{0}}\right)\times 100$$

where $A_{0}$ is the absorbance of the control and $A_{1}$ is the absorbance of the sample.

### 2.5 Metal‐Chelating Activity

Iron (Fe^2+^) metal‐chelating activity was determined following the method of Decker and Welch (1990). Extracts at concentrations of 25–400 μg/mL were prepared, and 1 mL of each extract was mixed with 3.7 mL deionized water. Then, 0.1 mL of 2 mM FeCl_2_ solution was added and incubated for 30 min.

Subsequently, 0.2 mL of 5 mM ferrozin solution was added to initiate the reaction. After 25 min at room temperature, absorbance was measured at 562 nm. Lower absorbance values indicated higher metal‐chelating capacity.

Chelating activity was compared with EDTA at the same concentrations. The percentage of metal chelation was calculated using the formula:

$$\text{Metal}‐\text{Chelating Activity}\;\left(\%\right)=\left(\frac{A_{0}-A_{1}}{A_{0}}\right)\times 100$$

where $A_{0}$ is the absorbance of the control and $A_{1}$ is the absorbance of the sample.

IC_50_ values were calculated by linear interpolation from concentration–response data. Dose–response relationships were evaluated using sigmoidal regression analysis.

### 2.6 *Drosophila melanogaster* Stock Culture and Experimental Conditions

*D. melanogaster* Oregon R (wild‐type) strain was used as the model organism in this study. The Oregon R strain was selected because it is a well‐characterized and widely used reference wild‐type background in Drosophila aging, toxicity, and oxidative stress research, facilitating comparison with previously published studies (Ashburner and Roote 2007; Fidan and Ayar 2022). The culture medium was prepared by adding 60 g sugar and 9 g agar to 440 mL boiling distilled water. Separately, 50 g cornmeal and 19 g yeast were dissolved in 125 mL distilled water and then mixed with the agar solution. Finally, 4.5 mL propionic acid was added as an antimicrobial agent, and the medium was dispensed into sterile glass culture bottles (Piper and Partridge 2016).

To obtain age‐synchronized individuals, virgin females emerging within 6–8 h after eclosion were collected and crossed with 3–5‐day‐old males. Third‐instar larvae (72 ± 4 h after the second crossing) were used for all experiments (Altun et al. 2011). Adult flies were counted under CO_2_ anesthesia using a stereomicroscope.

### 2.7 Toxicity and Lifespan Assays

Extracts from the aerial (leaves and flowers) and belowground (bulb and roots) parts of *G. elwesii* were prepared using three solvents: ethanol, methanol, and water. Four concentrations were tested: 1, 0.5, 0.25, and 0.125 μg/mL. These concentrations represent final concentrations in the culture medium. Stock solutions were prepared in 50% (v/v) DMSO and diluted 100‐fold with distilled water to achieve the desired final concentrations, resulting in a final DMSO concentration of 0.5% (v/v) in all treatment groups. The solvent control group received 0.5% (v/v) DMSO without extract.

Experimental groups consisted of: (i) negative control (medium + distilled water), (ii) solvent control (medium + 0.5% DMSO, v/v), (iii) aerial part extracts (12 groups), and (iv) belowground part extracts (12 groups), totalling 26 groups. For each treatment, 1.5 g instant medium (Carolina Formula 4–24) and 5 mL of the corresponding extract solution were added to sterile 100 mL glass bottles. One hundred third‐instar larvae were introduced into each bottle. All bottles were maintained at 25°C ± 1°C, 40%–60% relative humidity, and in darkness.

Third‐instar larvae were exposed to extracts throughout the feeding larval stage until pupation (approximately 48 h). Pupae developed on the same medium until adult eclosion (approximately 96 h post‐pupation), although direct feeding ceases at pupation. Thus, total developmental exposure spanned approximately 144 h (~6 days) from larval application to adult emergence. Emerging adults were separated by sex, and only males were used for lifespan assays to minimize variability related to female reproductive activity and associated metabolic differences (Magwere et al. 2004).

Adult males were transferred to fresh medium every 3 days. Mortality was recorded twice per week, and surviving individuals were counted until all flies had died. Lifespan data were used to construct Kaplan–Meier survival curves (Linford et al. 2013; Piper and Partridge 2016).

### 2.8 Biochemical Analyses (TAS, TOS, and OSI)

Based on the results of larval mortality and lifespan assays, 0.25 μg/mL was selected as the standard concentration for biochemical analyses. This concentration was chosen because it did not cause excessive larval mortality, allowed adequate adult emergence, and produced measurable effects on lifespan, enabling meaningful assessment of oxidative stress biomarkers.

Ascorbic acid (ASC; L‐ascorbic acid, Sigma‐Aldrich) was used as a positive antioxidant control at 20 mM, a concentration commonly employed in *D. melanogaster* oxidative stress studies. The solvent control consisted of 0.5% (v/v) DMSO in medium, while the negative control consisted of medium prepared with distilled water only.

For each treatment group, newly emerged male flies (0–24 h post‐eclosion) were collected to ensure age matching. For biochemical assays, 10 male flies were pooled per biological replicate (*n* = 3 independent replicates), homogenized together, and analyzed collectively. Samples were homogenized at a ratio of 1:9 (0.1 g tissue: 0.9 mL) in 140 mmol/L KCl buffer using an ultrasonic homogenizer. Homogenates were maintained on ice throughout the procedure. Protein concentrations were not normalized; instead, an equal tissue mass (0.1 g per replicate) was used for all groups to ensure consistency. Homogenates were centrifuged at 7000 rpm for 5 min at +4°C, and the supernatants were stored at −80°C until analysis.

Total antioxidant status (TAS) was measured using a commercial kit (Rel Assay Diagnostics) based on the ABTS radical‐scavenging method, and results were expressed as mmol Trolox equivalent/L (Erel 2004). Total oxidant status (TOS) was determined using a commercial kit based on ferrous ion oxidation, and results were expressed as μmol H_2_O_2_ equivalent/L (Erel 2005). The oxidative stress index (OSI) was calculated as follows: OSI = (TOS/TAS) × 100.

### 2.9 Statistical Analysis

Data are presented as mean ± standard error (SE) of at least three independent replicates. Normality and homogeneity of variance were verified using Shapiro–Wilk and Levene's tests, respectively. Differences among groups were analyzed by one‐way ANOVA followed by Tukey's HSD post hoc test. Survival data were evaluated using Kaplan–Meier analysis and compared by the log‐rank test. LC_50_ values were estimated using nonlinear regression analysis, and correlations were assessed using Pearson's coefficient. Statistical analyses were performed using SPSS v25.0 and GraphPad Prism v9.0, with significance set at *p* < 0.05. A post hoc power analysis conducted using the G*Power framework, based on observed *F* statistics and *η* ^2^ values derived from one‐way ANOVA, indicated that the sample sizes employed in lifespan assays (*n* = 3 biological replicates of 100 individuals per group) were sufficient to detect the large effect sizes observed in the present study (Cohen's *f* = 3.48–6.64; *η* ^2^ = 0.924–0.978), with statistical power approaching 1.00 for the primary lifespan‐related endpoints.

## 3 Results

### 3.1 Total Phenolic and Flavonoid Contents

The total phenolic and flavonoid contents of aerial (APE) and belowground (BPE) extracts of *G. elwesii* are presented in Table 1. Significant differences were observed among extracts (*p* < 0.05).

**TABLE 1:** Total phenolic and flavonoid contents of *Galanthus elwesii* extracts.

Extract	Total phenolic (mg GAE/g)	Total flavonoid (mg QE/g)
**Aerial extracts (APE)**		
APE‐W	27.45 ± 2.11ᵇ	21.91 ± 0.46ᵇ
APE‐E	14.31 ± 0.45ᵈ	17.50 ± 0.90ᵈ
APE‐M	33.56 ± 1.12ᵃ	24.32 ± 1.45ᵃ
**Belowground extracts (BPE)**		
BPE‐W	31.11 ± 1.23ᵃ	23.32 ± 1.18ᵃ
BPE‐E	13.86 ± 0.38ᵈ	17.00 ± 0.49ᵈ
BPE‐M	20.09 ± 0.86ᶜ	18.55 ± 0.63ᶜ

*Note:* Values are expressed as mean ± SD (*n* = 3). Different superscript letters within the same column indicate significant differences according to one‐way ANOVA followed by Tukey's HSD test (*p* < 0.05).

Abbreviations: APE, aerial part extract; BPE, belowground part extract; E, ethanol extract; M, methanol extract; W, aqueous (water) extract.

Among aerial extracts, the methanolic extract (APE‐M) exhibited the highest total phenolic (33.56 ± 1.12 mg GAE/g) and flavonoid content (24.32 ± 1.45 mg QE/g), whereas the ethanolic extract (APE‐E) showed the lowest levels of both phytochemicals. Similarly, among belowground extracts, BPE‐W demonstrated relatively high phenolic (31.11 ± 1.23 mg GAE/g) and flavonoid content (23.32 ± 1.18 mg QE/g), while BPE‐E exhibited the lowest values.

Overall, methanolic and aqueous extracts generally contained higher phenolic and flavonoid concentrations than ethanolic extracts.

### 3.2 DPPH Radical‐Scavenging Activity

The DPPH radical‐scavenging activities of the extracts increased in a concentration‐dependent manner (Table 2). Belowground extracts generally exhibited stronger antioxidant activity than aerial extracts at all tested concentrations.

**TABLE 2:** DPPH radical‐scavenging activity (% inhibition) of aerial (APE) and belowground (BPE) extracts of *Galanthus elwesii* at different concentrations and their corresponding IC_50_ values.

Extract	25 μg/mL	50 μg/mL	100 μg/mL	200 μg/mL	400 μg/mL	IC_50_
APE‐W	47.76 ± 0.59^c^	53.59 ± 1.13^a^	58.52 ± 1.46^b^	61.86 ± 1.34^a^	71.86 ± 1.68^ab^	34.60 ± 1.17
APE‐E	39.85 ± 0.7^d^	44.15 ± 1.25^a^	55.82 ± 4.49^b^	61.51 ± 1.15^a^	65.84 ± 1.01^b^	75.98 ± 5.69
APE‐M	35.72 ± 0.8^d^	62.56 ± 21.9^a^	74.33 ± 4.35a	63.74 ± 23.31^a^	82.30 ± 1.00^a^	40.45 ± 7.62
BPE‐W	92.84 ± 0.48^a^	64.46 ± 0.93^a^	67.50 ± 0.70^ab^	71.37 ± 1.54^a^	77.34 ± 3.29^ab^	13.46 ± 0.03
BPE‐E	31.44 ± 7.7^d^	52.84 ± 6.91^a^	56.31 ± 2.49^b^	60.31 ± 11.3^a^	78.63 ± 1.99^ab^	48.86 ± 6.08
BPE‐M	56.65 ± 1.3^b^	66.11 ± 1.76^a^	74.19 ± 10.7^a^	82.17 ± 14.2^a^	86.86 ± 16.7^a^	22.07 ± 0.24

*Note:* Values are expressed as mean ± SD (*n* = 3). Different letters within the same column indicate significant differences according to one‐way ANOVA followed by Tukey's HSD test (*p* < 0.05).

Abbreviations: APE, aerial part extract; BPE, belowground part extract; E, ethanol extract; M, methanol extract; W, aqueous (water) extract.

At 400 μg/mL, BPE‐M showed the highest radical‐scavenging activity (86.86% ± 16.72%), followed by BPE‐W (77.34% ± 3.29%). Among aerial extracts, APE‐M exhibited the strongest activity (82.30% ± 1.00%), whereas APE‐E showed the lowest activity.

The IC_50_ values supported these findings. BPE‐W displayed the lowest IC_50_ value (13.46 ± 0.03 μg/mL), indicating the strongest antioxidant capacity, followed by BPE‐M (22.07 ± 0.24 μg/mL). In contrast, APE‐E exhibited the highest IC_50_ value (75.98 ± 5.69 μg/mL).

It should be noted that certain extract–concentration combinations, particularly APE‐M at intermediate concentrations (50 and 200 μg/mL), displayed relatively high inter‐replicate variability (CV = 35%–37%). This pattern was not observed at the lowest or highest concentrations tested for the same extract, suggesting that the variability was concentration‐dependent rather than indicative of generalized technical inconsistency. Such nonlinear concentration‐dependent responses may reflect the biochemical complexity of crude plant extracts containing multiple interacting phytochemical constituents with potentially synergistic or antagonistic effects. Importantly, the overall reproducibility of the assay was supported by the consistently low variability observed across most other extract–concentration combinations (CV < 5%).

A comparable non‐monotonic pattern was also observed for BPE‐W, where DPPH‐scavenging activity at 25 μg/mL exceeded that measured at 50 μg/mL. Likewise, larval mortality responses for APE‐E and APE‐W did not follow a strictly linear concentration‐dependent pattern across all tested doses. Such variability may reflect concentration‐dependent interactions among multiple phytochemical constituents present in crude plant extracts.

### 3.3 Metal‐Chelating Activity

The Fe^2+^ metal‐chelating activities of the extracts also showed concentration‐dependent increases (Table 3). Significant differences were observed among extracts and reference antioxidants (*p* < 0.05).

**TABLE 3:** Fe^2+^ metal‐chelating activity (%) of *G. elwesii* extracts and standards (mean ± SD, *n* = 3) and IC_50_ (mean ± SE).

Extract	25 μg/mL	50 μg/mL	100 μg/mL	200 μg/mL	400 μg/mL	IC_50_ (μg/mL)
APE‐W	23.06 ± 1.08ᶠ	34.05 ± 1.14ᵍ	46.64 ± 0.98ᵈ	57.51 ± 3.62ᵈ	65.95 ± 0.57ᶜ	131.46 ± 6.05
APE‐E	4.83 ± 0.22ᵍ	9.70 ± 0.27ʰ	32.91 ± 0.74ᵉ	70.37 ± 0.66ᶜ	71.76 ± 1.49ᵇ	145.61 ± 0.64
APE‐M	35.85 ± 2.09ᵉ	40.44 ± 0.08ᶠ	49.26 ± 1.95ᵈ	56.26 ± 2.53ᵈ	70.16 ± 4.73ᶜ	111.23 ± 10.16
BPE‐W	58.55 ± 1.68ᶜ	74.58 ± 3.13ᵇ	76.11 ± 1.30ᵇ	77.46 ± 3.42ᵇ	82.11 ± 4.16ᵇ	21.35 ± 0.29
BPE‐E	57.91 ± 1.44ᶜ	59.30 ± 1.69ᵈ	63.18 ± 3.11ᶜ	75.48 ± 0.22ᵇ	87.32 ± 5.64ᵃ	21.59 ± 0.26
BPE‐M	47.21 ± 0.65ᵈ	53.83 ± 2.06ᵉ	60.11 ± 2.14ᶜ	60.78 ± 2.62ᵈ	77.99 ± 2.01ᵇ	35.78 ± 1.72
EDTA	70.85 ± 1.57ᵇ	72.32 ± 1.89ᵇ	79.47 ± 2.51ᵃ	81.09 ± 4.33ᵇ	96.93 ± 5.02ᵃ	17.64 ± 0.18
BHT	77.00 ± 1.35ᵃ	81.25 ± 3.29ᵃ	83.09 ± 0.99ᵃ	94.20 ± 3.44ᵃ	97.50 ± 1.69ᵃ	16.24 ± 0.14
Trolox	54.27 ± 2.95ᶜ	67.84 ± 0.03ᶜ	74.25 ± 2.15ᵇ	81.53 ± 2.01ᵇ	95.45 ± 3.96ᵃ	23.05 ± 0.59

*Note:* Values are presented as mean ± SD (*n* = 3). Different superscript letters within the same column indicate significant differences (one‐way ANOVA followed by Tukey's HSD, *p* < 0.05).

Abbreviations: APE, aerial part extract; BPE, belowground part extract; E, ethanol extract; M, methanol extract; W, aqueous (water) extract.

Among plant extracts, belowground samples demonstrated markedly higher chelating capacity than aerial extracts. At 400 μg/mL, BPE‐E exhibited the highest chelating activity (87.32% ± 5.64%), followed by BPE‐W (82.11% ± 4.16%).

IC_50_ analysis revealed that BPE‐W and BPE‐E had the lowest IC_50_ values (21.35 ± 0.29 and 21.59 ± 0.26 μg/mL, respectively), indicating strong metal chelation capacity. In contrast, aerial extracts showed substantially higher IC_50_ values, particularly APE‐E (145.61 ± 0.64 μg/mL).

Among reference antioxidants, BHT demonstrated the strongest chelating activity, followed by EDTA and Trolox (Table 3).

### 3.4 Correlation Analysis

**FIGURE 1:**
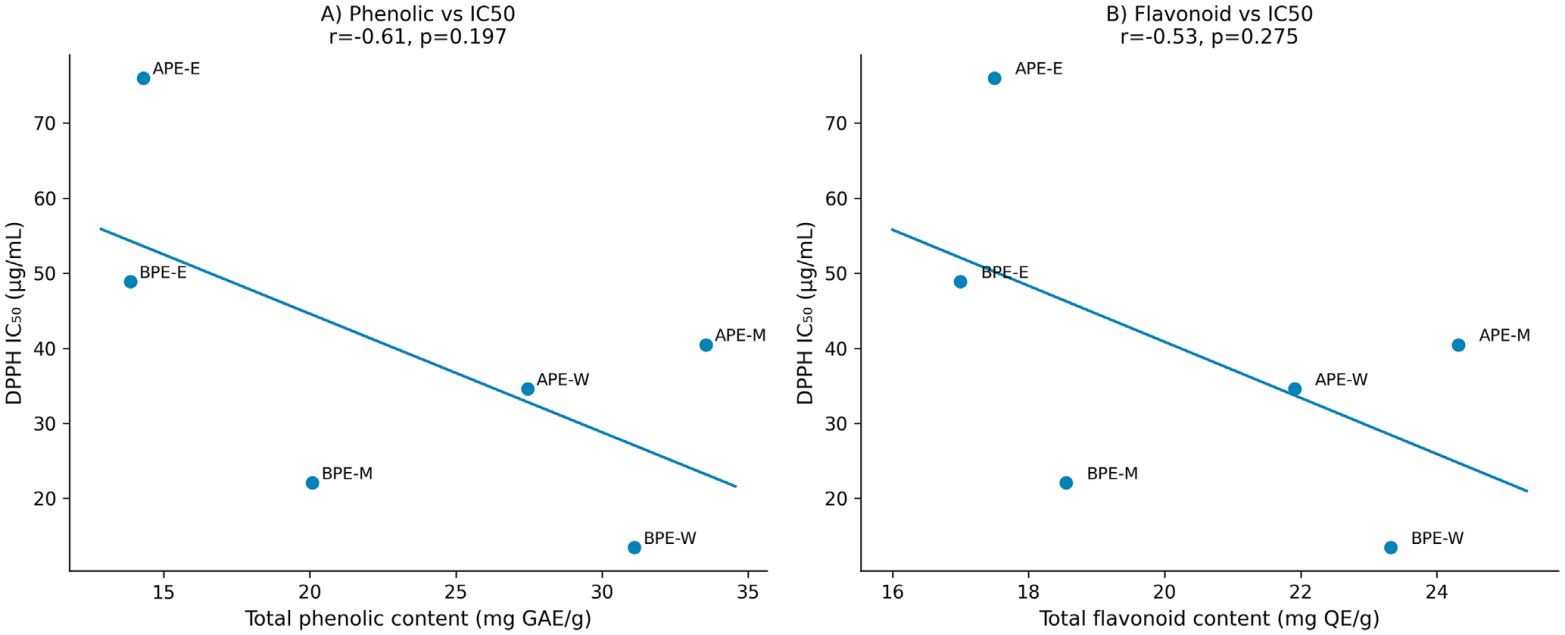
Correlation between phytochemical content and antioxidant activity of *Galanthus elwesii* extracts. (A) Relationship between total phenolic content and DPPH IC_50_ values. (B) Relationship between total flavonoid content and DPPH IC_50_ values. Negative correlations indicate that higher phytochemical content is associated with stronger radical‐scavenging activity.

Correlation analysis revealed a moderate negative relationship between total phenolic content and DPPH IC_50_ values (*r* = −0.61) and a similar trend for flavonoid content (*r* = −0.53); however, neither correlation reached statistical significance (*p* > 0.05, Figure 1). These findings should therefore be interpreted cautiously and do not provide statistically significant evidence for direct associations between phenolic/flavonoid content and antioxidant activity within the present dataset.

### 3.5 In Vivo Toxicity and Effects on Lifespan

#### 3.5.1 Larval Mortality and Developmental Toxicity

The dose‐dependent toxic effects of aerial (APE) and belowground (BPE) extracts of *G. elwesii* , prepared using ethanol (E), methanol (M), and water (W), were evaluated in *D. melanogaster* third‐instar larvae. Larvae were continuously exposed to final medium concentrations of 1, 0.5, 0.25, and 0.125 μg/mL from the third‐instar stage until adult emergence. All assays were performed in three independent biological replicates (*n* = 3; 100 larvae per replicate). Larval mortality varied significantly in treatment‐, solvent‐, and dose‐dependent manner (one‐way ANOVA followed by Tukey's HSD, *p* < 0.05) (Table 4).

**TABLE 4:** Larval mortality of *Drosophila melanogaster* following exposure to aerial and belowground extracts with estimated LC_50_ values.

Treatment group	Mortality (%) (mean ± SE)	Estimated LC_50_ (μg/mL)
Control (distilled water)	4.0 ± 0.6^a^	—
DMSO control (0.5%)	12.0 ± 0.6^b^	—
APE‐E 1	9.0 ± 0.6^a^	1.04
APE‐E 0.5	22.0 ± 0.9^c^	
APE‐E 0.25	29.0 ± 0.9^c^	
APE‐E 0.125	10.0 ± 0.6^a^	
APE‐M 1	55.0 ± 1.3^f^	~0.60 (approx.)^1^
APE‐M 0.5	48.0 ± 1.3^e^	
APE‐M 0.25	49.0 ± 1.3^e^	
APE‐M 0.125	44.0 ± 1.3^e^	
APE‐W 1	30.0 ± 0.9^c^	0.51
APE‐W 0.5	39.0 ± 1.3^d^	
APE‐W 0.25	47.0 ± 1.3^e^	
APE‐W 0.125	42.0 ± 1.3^e^	
**Belowground part extract (BPE) (μg/mL)**		
BPE‐E 1	14.0 ± 0.6^b^	1.07
BPE‐E 0.5	22.0 ± 0.9^c^	
BPE‐E 0.25	14.0 ± 0.6^b^	
BPE‐E 0.125	17.0 ± 0.9^b^	
BPE‐M 1	61.0 ± 1.3^f^	0.51
BPE‐M 0.5	30.0 ± 0.9^c^	
BPE‐M 0.25	6.0 ± 0.6^a^	
BPE‐M 0.125	16.0 ± 0.9^b^	
BPE‐W 1	8.0 ± 0.6^a^	0.12
BPE‐W 0.5	7.0 ± 0.6^a^	
BPE‐W 0.25	8.0 ± 0.6^a^	
BPE‐W 0.125	5.0 ± 0.6^a^	

*Note:* Values represent the mean percentage of larval mortality ± standard error (SE) from three independent replicates (*n* = 3). Each replicate consisted of 100 larvae per treatment group. Differences among treatment groups were analyzed by one‐way ANOVA followed by Tukey's HSD post hoc test (*p* < 0.05). Lowercase letters indicate the results of multiple comparisons; treatments sharing the same letter are not significantly different. LC_50_ values were estimated by fitting a four‐parameter sigmoidal (logistic) dose–response model to mean larval mortality data using nonlinear regression.

Abbreviations: APE, aerial part extract; BPE, belowground part extract; DMSO, dimethyl sulfoxide; E, ethanol extract; M, methanol extract; SE, standard error; W, aqueous (water) extract.

^1^ For APE‐M, LC_50_ was approximated by interpolation from mean mortality values because the dose–response pattern was not sufficiently monotonic to permit reliable nonlinear regression.

**FIGURE 2:**
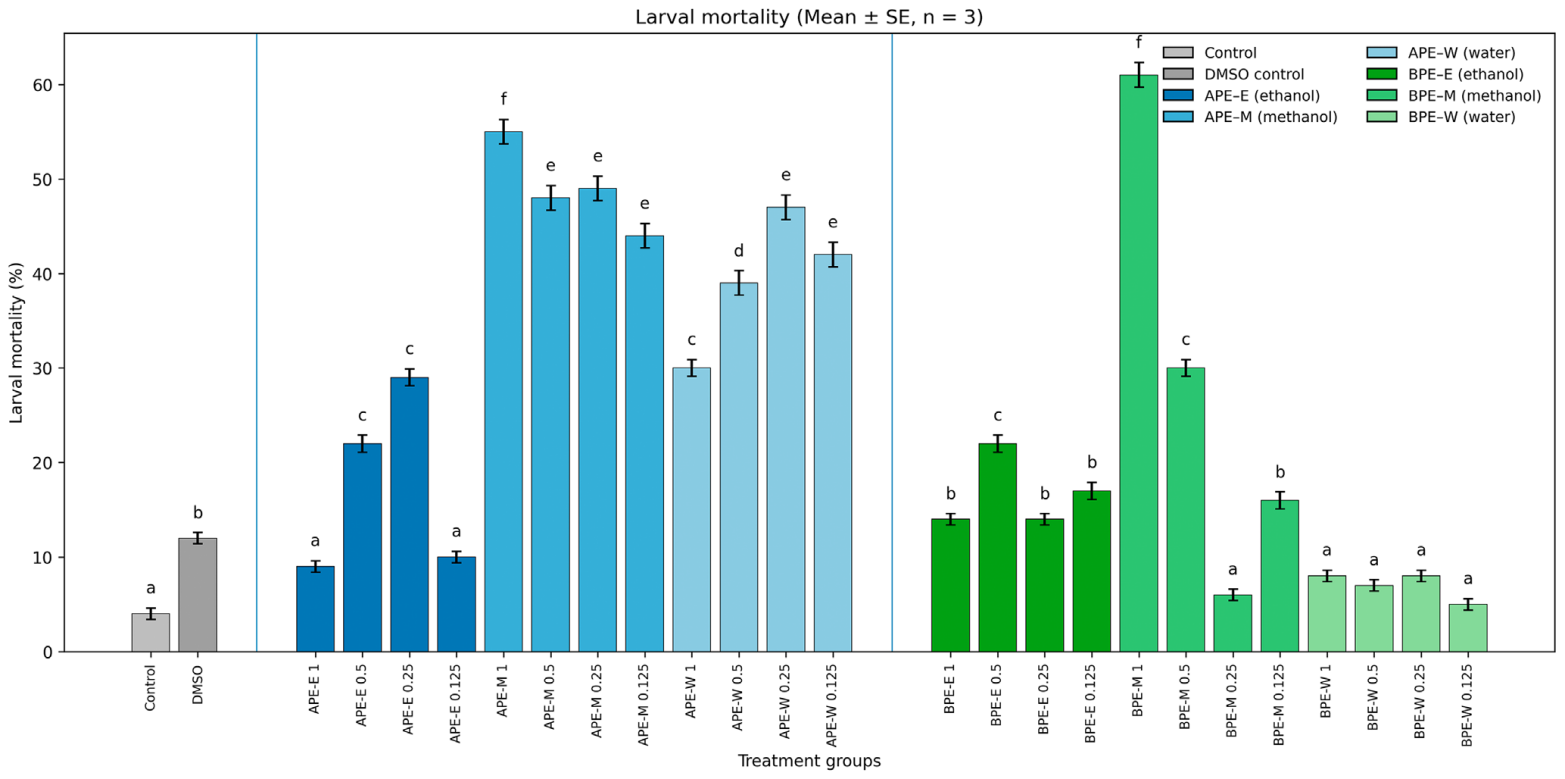
Larval mortality of *Drosophila melanogaster* exposed to aerial (APE) and belowground (BPE) extracts of *Galanthus elwesii* . Bars represent mean ± SE from three independent replicates (*n* = 100 larvae per replicate). Different lowercase letters indicate significant differences (one‐way ANOVA followed by Tukey's HSD, *p* < 0.05).

Overall, methanolic extracts (APE‐M and BPE‐M) exhibited the highest toxicity among all treatments. Mortality was significantly elevated relative to the distilled water control in APE‐M 1 μg/mL (55%) and BPE‐M 1 μg/mL (61%) groups (*p* < 0.05), indicating strong concentration‐dependent lethality at the highest dose (Table 4; Figure 2). In contrast, at lower concentrations, BPE‐M showed a marked reduction in mortality (6% at 0.25 μg/mL), suggesting a nonlinear response.

Ethanolic extracts (APE‐E and BPE‐E) displayed moderate toxicity. In APE‐E, mortality remained low at 1 μg/mL (9%) but increased at 0.5–0.25 μg/mL (22%–29%), whereas BPE‐E induced comparatively lower mortality across all doses (14%–22%) (Table 4; Figure 2).

Aqueous extracts (APE‐W and BPE‐W) were the least toxic. Mortality in BPE‐W treatments (5%–8%) did not differ significantly from the control (*p* > 0.05), whereas APE‐W caused a moderate increase in mortality at 1 μg/mL (30%) (Table 4; Figure 2).

**FIGURE 3:**
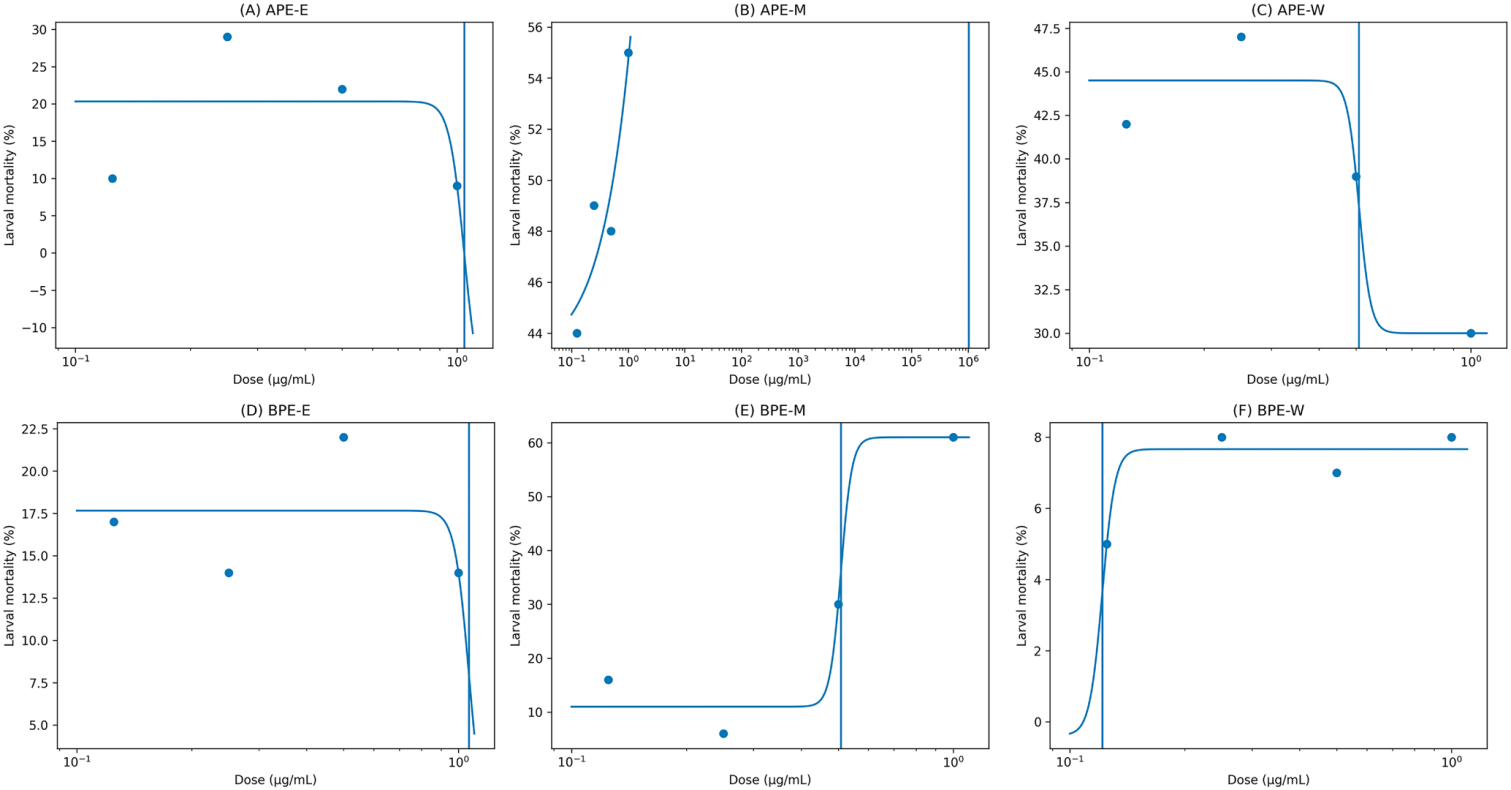
Dose–response curves for larval mortality of *Drosophila melanogaster* following exposure to aerial and belowground extracts. Points represent mean mortality (*n* = 3 replicates; 100 larvae per replicate). Curves depict fitted four‐parameter sigmoidal (logistic) models; vertical lines indicate estimated LC_50_ values. For APE‐M, LC50 (~0.60 μg/mL) was approximated by interpolation due to a non‐monotonic dose–response. Panels: (A) APE‐E, (B) APE‐M, (C) APE‐W, (D) BPE‐E, (E) BPE‐M, (F) BPE‐W.

Sigmoidal dose–response modeling of mean larval mortality allowed estimation of LC_50_ values for most extracts (Figure 3). Estimated LC_50_ values were 1.04 μg/mL for APE‐E, 0.51 μg/mL for APE‐W, 1.07 μg/mL for BPE‐E, and 0.51 μg/mL for BPE‐M. For BPE‐W, however, mortality rates remained consistently low across all tested concentrations, preventing reliable estimation of a biologically meaningful LC_50_ value using sigmoidal dose–response modeling (Table 4; Figure 3).

Collectively, these findings demonstrate that solvent type and plant part strongly influenced larval toxicity, with methanolic extracts showing the highest toxicity and aqueous belowground extracts exhibiting minimal lethality.

#### 3.5.2 Lifespan Effects of Aerial Parts Extracts (APE)

Lifespan parameters of male *D. melanogaster* exposed to APE extracts are summarized in Table 5. Distilled water and 0.5% DMSO controls did not differ significantly in mean or maximum lifespan (*p* > 0.05, log‐rank test).

**TABLE 5:** Lifespan parameters of *Drosophila melanogaster* treated with APE extracts.

Extract	Group	Maximum lifespan (days) ± SE	Mean lifespan (days) ± SE	Median lifespan (KM interval, days)	Estimated median (days)	% change vs. control (mean)
—	Control	77 ± 1.36ᵇ	52 ± 1.10ᵃ	33–40	36.5	—
—	DMSO (0.5%)	71 ± 0.95ᵃ	51 ± 0.58ᵃ	26–33	31.7	−1.9%
APE‐E	1 μg/mL	79 ± 1.32ᵇ	57 ± 0.90ᵃ	47–54	53.2	+9.6%
APE‐E	0.5 μg/mL	72 ± 0.88ᵃ	44 ± 1.42ᵇ	26	26.0	−15.4%
APE‐E	0.25 μg/mL	76 ± 1.32ᵇ	53 ± 0.83ᵃ	40–47	40.8	+1.9%
APE‐E	0.125 μg/mL	76 ± 0.98ᵇ	47 ± 0.41ᵇ	47–54	48.6	−9.6%
APE‐M	1 μg/mL	62 ± 1.22ᶜ	34 ± 0.49ᶜ	7–12	11.2	−34.6%
APE‐M	0.5 μg/mL	62 ± 1.33ᶜ	43 ± 0.82ᵇ	12–19	14.8	−17.3%
APE‐M	0.25 μg/mL	66 ± 1.16ᶜ	40 ± 0.35ᵇ	33–40	34.4	−23.1%
APE‐M	0.125 μg/mL	54 ± 0.78ᵈ	40 ± 1.12ᵇ	19–26	24.7	−23.1%
APE‐W	1 μg/mL	69 ± 0.63ᵃ	47 ± 0.45ᵇ	26–33	28.7	−9.6%
APE‐W	0.5 μg/mL	76 ± 1.68ᵇ	41 ± 1.12ᵇ	40–47	44.0	−21.2%
APE‐W	0.25 μg/mL	74 ± 0.98ᵇ	54 ± 1.53ᵃ	40–47	42.3	+3.8%
APE‐W	0.125 μg/mL	74 ± 1.10ᵇ	43 ± 0.97ᵇ	40–47	42.6	−17.3%

*Note:* Different superscript letters within the same column indicate statistically significant differences (*p* < 0.05). Median lifespan was estimated from Kaplan–Meier curves based on pooled mean survivorship across three independent replicates (*n* = 100 per replicate). Estimated median assumes uniform mortality within each observation interval. Survival was recorded every 3 days until all individuals died. Control and DMSO groups were shared across both APE and BPE experiments and were conducted simultaneously; therefore, statistical groupings are directly comparable between Tables 5 and 6.

Abbreviations: APE‐E, ethanolic aerial parts extract; APE‐M, methanolic aerial parts extract; APE‐W, water aerial parts extract; SE, standard error.

Among APE‐E treatments, 1 μg/mL significantly increased mean lifespan (+9.6%), whereas 0.5 and 0.125 μg/mL significantly reduced it (−15.4% and −9.6%, respectively). APE‐M was generally detrimental, with 1 μg/mL markedly shortening lifespan (−34.6%), while lower doses partially mitigated this effect but remained significantly different from control. APE‐W exhibited a nonlinear response; 0.25 μg/mL maintained mean lifespan comparable to control (+3.8%), whereas other doses reduced survival.

**FIGURE 4:**
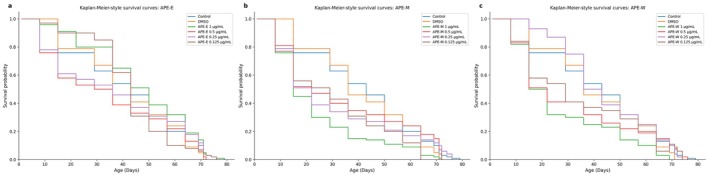
Kaplan–Meier survival curves of male *Drosophila melanogaster* treated with aerial parts extracts (APE). Survival was recorded every 3 days until all individuals died. Curves represent pooled data from three independent biological replicates (*n* = 100 males per replicate). Statistical comparisons with the distilled water control were performed using the log‐rank (Mantel–Cox) test.

These patterns were consistent with Kaplan–Meier survival curves (Figure 4), which were compared using the log‐rank (Mantel–Cox) test.

It should also be noted that the LC_50_ value for APE‐M could not be derived from standard sigmoidal regression due to a non‐monotonic dose–response relationship observed across the tested concentrations. This pattern may reflect the biochemical complexity of crude plant extracts containing multiple bioactive constituents with potentially opposing biological effects. Therefore, the LC_50_ value was estimated by linear interpolation and should be interpreted with appropriate caution.

#### 3.5.3 Lifespan Effects of BPE (Belowground Parts Extracts)

As summarized in Table 6, belowground parts extracts (BPE) also influenced lifespan in a dose‐dependent manner. BPE‐E showed modest effects, with 0.25 μg/mL significantly increasing mean lifespan (+5.8%).

**TABLE 6:** Lifespan parameters of *Drosophila melanogaster* treated with BPE extracts.

Extract	Group	Maximum lifespan (days) ± SE	Mean lifespan (days) ± SE	Median lifespan (KM interval, days)	Estimated median (days)	% change vs. control (mean)
—	Control	77 ± 1.36ᵃ	52 ± 1.10ᵃ	33–40	36.5	—
—	DMSO (0.5%)	71 ± 0.95ᵇ	51 ± 0.58ᵃ	26–33	31.7	−1.9%
BPE‐E	1 μg/mL	68 ± 0.93ᵇ	49 ± 1.83ᵃ	33–40	36.0	−5.8%
BPE‐E	0.5 μg/mL	73 ± 1.36ᵃᵇ	49 ± 1.14ᵃ	40–47	41.8	−5.8%
BPE‐E	0.25 μg/mL	74 ± 0.77ᵃ	55 ± 0.78ᵃ	40–47	46.0	+5.8%
BPE‐E	0.125 μg/mL	68 ± 1.56ᵇ	49 ± 0.44ᵃ	33–40	39.2	−5.8%
BPE‐M	1 μg/mL	68 ± 1.33ᵇ	35 ± 1.14ᶜ	7–12	10.5	−32.7%
BPE‐M	0.5 μg/mL	73 ± 1.66ᵃᵇ	47 ± 0.68ᵃᵇ	40–47	45.2	−9.6%
BPE‐M	0.25 μg/mL	78 ± 1.37ᵃ	57 ± 0.47ᵃ	54–58	57.6	+9.6%
BPE‐M	0.125 μg/mL	78 ± 0.65ᵃ	55 ± 1.11ᵃ	66–68	67.2	+5.8%
BPE‐W	1 μg/mL	68 ± 1.16ᵇ	51 ± 1.42ᵃ	33–40	39.1	−1.9%
BPE‐W	0.5 μg/mL	77 ± 1.28ᵃ	54 ± 0.33ᵃ	54–58	55.5	+3.8%
BPE‐W	0.25 μg/mL	79 ± 1.19ᵃ	60 ± 0.59ᵈ	62–66	63.7	+15.4%
BPE‐W	0.125 μg/mL	68 ± 0.63ᵇ	49 ± 1.14ᵃ	33–40	37.8	−5.8%

*Note:* Different superscript letters within the same column indicate statistically significant differences (*p* < 0.05). Median lifespan was estimated from Kaplan–Meier curves based on pooled mean survivorship across three independent replicates (*n* = 100 per replicate). Estimated median assumes uniform mortality within each observation interval. Survival was recorded every 3 days until all individuals died. Control and DMSO groups were shared across both APE and BPE experiments and were conducted simultaneously; therefore, statistical groupings are directly comparable between Tables 5 and 6.

Abbreviations: BPE‐E, ethanolic belowground parts extract; BPE‐M, methanolic belowground parts extract; BPE‐W, water belowground parts extract; SE, standard error.

**FIGURE 5:**
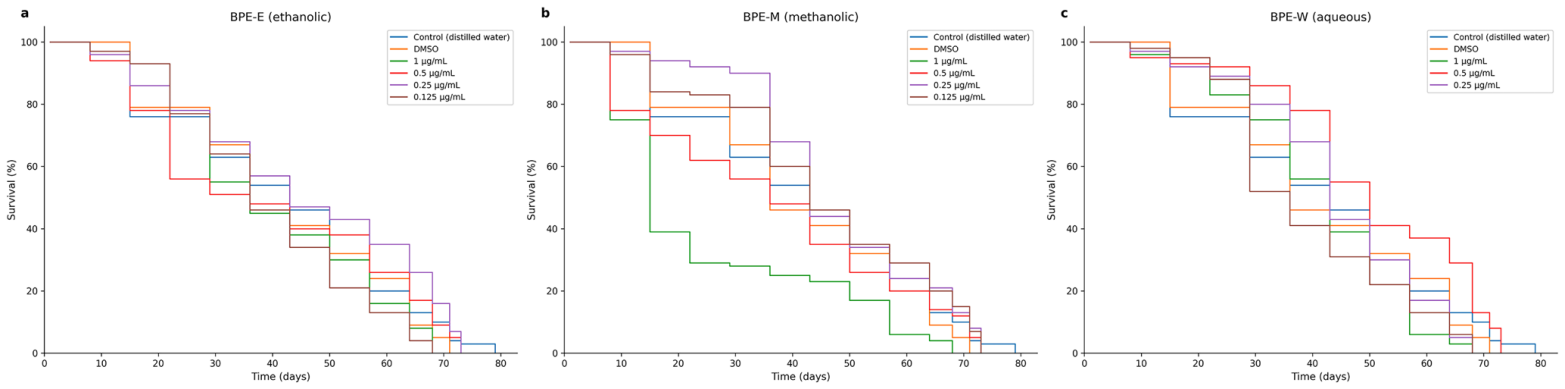
Kaplan–Meier survival curves of male *Drosophila melanogaster* treated with belowground parts extracts (BPE). Survival was recorded every 3 days until the death of the last individual. Curves represent pooled data from three independent biological replicates (*n* = 100 males per replicate). Statistical comparisons with the distilled water control were performed using the log‐rank (Mantel–Cox) test.

BPE‐M displayed a biphasic dose‐dependent pattern: 1 μg/mL was strongly detrimental (−32.7%), whereas 0.25 and 0.125 μg/mL significantly prolonged lifespan (+9.6% and +5.8%, respectively). BPE‐W produced the most pronounced extension at 0.25 μg/mL (+15.4%), while 0.5 μg/mL also improved survival. These patterns were reflected in the Kaplan–Meier curves (log‐rank test, *p* < 0.05) (Figure 5).

Overall, BPE extracts exhibited a broader dynamic range than APE, showing toxicity at high doses but lifespan extension at lower doses, particularly for methanolic and aqueous extracts.

### 3.6 Effects on Oxidative Stress Biomarkers

**FIGURE 6:**
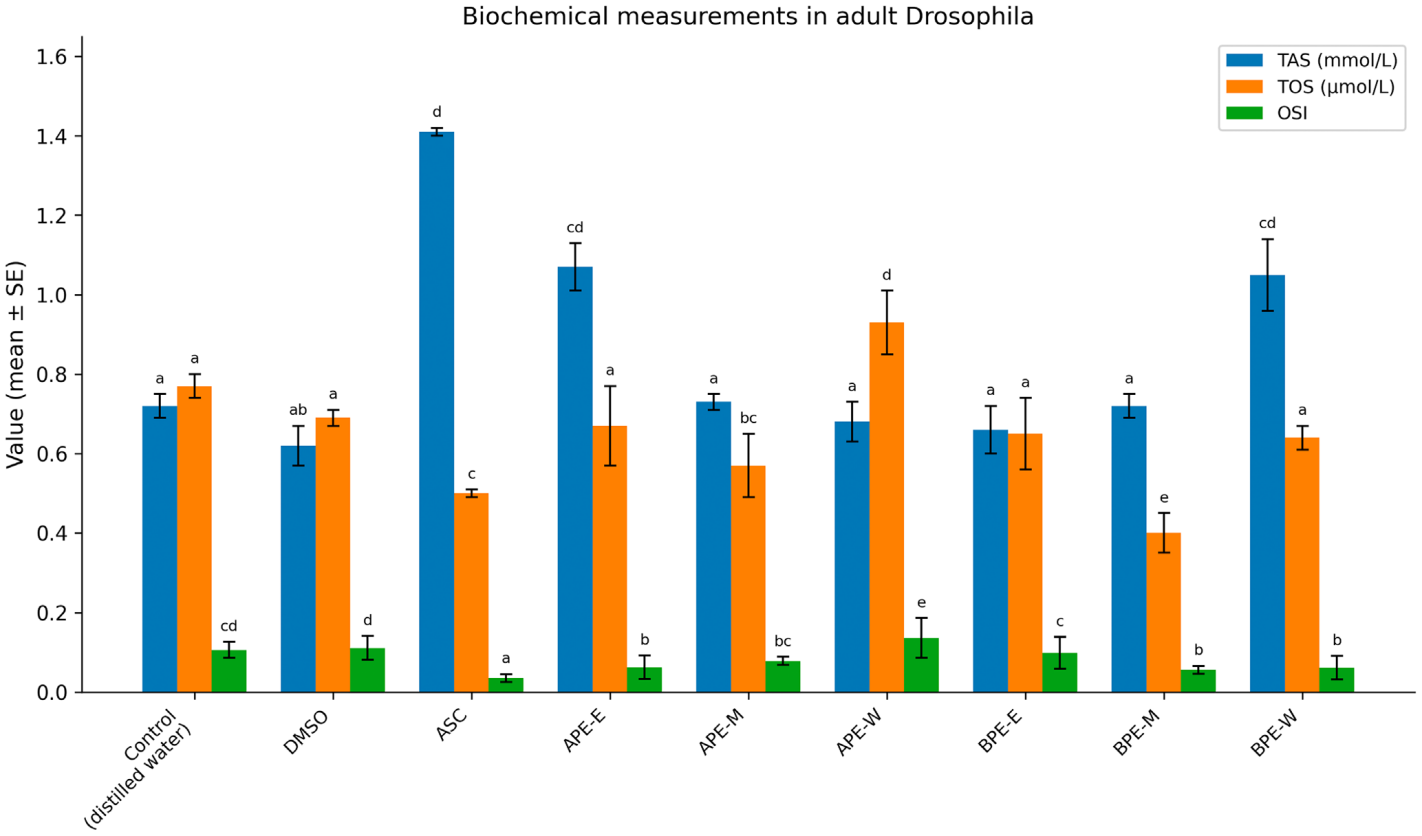
Oxidative stress biomarkers (TAS, TOS, and OSI) in adult *Drosophila melanogaster* following exposure to *Galanthus elwesii* extracts. Newly emerged males (0–24 h post‐eclosion) were analyzed after continuous exposure to 0.25 μg/mL of each extract throughout development. Data represent mean ± SE from three independent biological replicates (10 pooled males per replicate). Differences among groups were analyzed by one‐way ANOVA followed by Tukey's HSD post hoc test (*p* < 0.05).

TAS, TOS, and OSI values for all groups are presented in Table 7 and Figure 6. Significant differences among treatments were detected by one‐way ANOVA followed by Tukey's HSD test (*p* < 0.05). Ascorbic acid (ASC) (20 mM) displayed a markedly enhanced antioxidant profile, characterized by significantly higher TAS and significantly lower TOS and OSI compared with both control and DMSO groups (0.5% v/v).

**TABLE 7:** Oxidative status parameters (TAS, TOS, OSI) in *Drosophila melanogaster* after treatment with aerial and belowground plant extracts.

Experimental group	TAS (mmol Trolox eq/L) ± SE	TOS (μmol H_2_O_2_ eq/L) ± SE	OSI ± SE
Control (distilled water)	0.72 ± 0.03^a^	0.77 ± 0.03^a^	0.106 ± 0.02^cd^
DMSO (0.5%)	0.62 ± 0.05^ab^	0.69 ± 0.02^a^	0.111 ± 0.03^d^
ASC (20 mM)	1.41 ± 0.01^d^	0.50 ± 0.01^c^	0.035 ± 0.01^a^
APE‐E (0.25 μg/mL)	1.07 ± 0.06^cd^	0.67 ± 0.10^a^	0.062 ± 0.03^b^
APE‐M (0.25 μg/mL)	0.73 ± 0.02^a^	0.57 ± 0.08^bc^	0.078 ± 0.01^bc^
APE‐W (0.25 μg/mL)	0.68 ± 0.05^a^	0.93 ± 0.08^d^	0.136 ± 0.05^e^
BPE‐E (0.25 μg/mL)	0.66 ± 0.06^a^	0.65 ± 0.09^a^	0.098 ± 0.04^c^
BPE‐M (0.25 μg/mL)	0.72 ± 0.03^a^	0.40 ± 0.05^e^	0.056 ± 0.01^b^
BPE‐W (0.25 μg/mL)	1.05 ± 0.09^cd^	0.64 ± 0.03^a^	0.061 ± 0.03^b^

*Note:* Different superscript letters within the same column indicate statistically significant differences (*p* < 0.05). For subsequent biochemical analyses, 0.25 μg/mL was selected as the standard experimental concentration.

Abbreviations: APE‐E/M/W, ethanolic, methanolic, and water aerial parts extracts; ASC, ascorbic acid; BPE‐E/M/W, ethanolic, methanolic, and water belowground parts extracts; DMSO, dimethyl sulfoxide; OSI, oxidative stress index; SE, standard error; TAS, total antioxidant status; TOS, total oxidant status.

Among aerial parts extracts (APE), the ethanolic extract (APE‐E) significantly increased TAS relative to control while maintaining TOS at control‐like levels and reducing OSI, indicating an overall improvement in oxidative balance. In contrast, the water extract (APE‐W) significantly elevated both TOS and OSI compared with control, reflecting increased oxidative stress. The methanolic extract (APE‐M) did not significantly alter TAS but moderately reduced TOS and OSI relative to control.

For belowground parts extracts (BPE), the methanolic extract (BPE‐M) exerted the most pronounced antioxidant‐related effects, significantly decreasing TOS and OSI without reducing TAS. Similarly, BPE‐W significantly increased TAS while keeping TOS comparable to control, resulting in a lower OSI. BPE‐E did not significantly modify TAS or TOS compared with control but led to a moderate reduction in OSI.

Overall, ASC, APE‐E, BPE‐M, and BPE‐W significantly reduced OSI and/or TOS and increased or maintained TAS, whereas APE‐W significantly increased TOS and OSI compared with the control.

## 4 Discussion

This study provides a comprehensive integrated evaluation of the organ‐dependent phytochemical profile and redox‐modulatory effects of *G. elwesii* extracts by combining in vitro antioxidant assays with in vivo lifespan and oxidative stress analyses in *Drosophila melanogaster* . The findings indicate that the biological activity of the extracts is strongly influenced by plant organ, solvent polarity, and dose, with belowground extracts (BPE) generally exhibiting stronger antioxidant capacity and more pronounced lifespan‐modulating effects than aerial extracts (APE). These results extend previous phytochemical studies on *Galanthus* species by showing that their biological effects cannot be explained solely by direct radical‐scavenging capacity but instead involve modulation of endogenous redox homeostasis and adaptive stress responses. The organ‐ and solvent‐dependent bioactivity patterns observed in the present study may be consistent with previously reported compositional variation between aerial and belowground parts of *G. elwesii* under different extraction conditions (Ay et al. 2020, 2023). However, because direct chromatographic characterization was not performed within the current study, these associations should be interpreted cautiously.

A major outcome of this study is the pronounced organ‐dependent variation in phytochemical composition, where BPE extracts showed higher phenolic and flavonoid contents than APE extracts. In particular, BPE‐W and APE‐M exhibited the highest phenolic concentrations, whereas ethanolic extracts (APE‐E and BPE‐E) consistently contained the lowest levels. This pattern aligns with previous evidence indicating that geophyte storage organs may contain relatively high levels of secondary metabolites and other bioactive constituents (Berkov et al. 2011; Ay et al. 2020; Aydın and Mammadov 2022). The differential solvent efficacy observed reflects the well‐established principle that phenolic compounds exhibit variable polarity and are most efficiently extracted by intermediate‐polarity solvents such as methanol, whereas aqueous extraction favors hydrophilic glycosylated metabolites (Ncube et al. 2008; Benedec et al. 2018). The strong radical‐scavenging and metal‐chelating activities observed particularly in BPE‐W and BPE‐M support the interpretation that belowground tissues harbor metabolite pools enriched in phenolics and alkaloids with potent antioxidant potential (Berkov et al. 2011; Georgiev et al. 2024). The moderate negative correlations observed between phenolic content and DPPH IC_50_ (*r* = −0.61) and flavonoid content and DPPH IC_50_ (*r* = −0.53) did not reach statistical significance (*p* > 0.05) and therefore cannot be interpreted as confirmatory evidence of direct associations between phytochemical content and antioxidant activity within the present dataset. Nevertheless, the directional consistency of these trends with previous studies on plant‐derived polyphenols (Pisoschi and Pop 2015) may indicate that phenolic compounds could contribute, at least partially, to the antioxidant properties of *G. elwesii* extracts. However, this interpretation remains speculative and requires validation through larger sample sets and chromatographically characterized fractions.

A notable finding of the present study is the strong solvent‐dependent variation in developmental toxicity. Methanolic extracts, particularly APE‐M and BPE‐M, exhibited the highest larval mortality, whereas aqueous extracts, especially BPE‐W, showed minimal toxicity despite high antioxidant activity. This paradox may partially reflect co‐extraction of cytotoxic Amaryllidaceae alkaloids such as lycorine and narciclasine in methanolic fractions, compounds known to inhibit protein synthesis and exert potent antiproliferative effects (Berkov et al. 2011; He et al. 2015). Conversely, aqueous extraction preferentially recovers hydrophilic polyphenols while excluding lipophilic alkaloids, explaining the favorable safety profile of BPE‐W. The elevated toxicity observed at high concentrations also reflects the dual nature of plant secondary metabolites, which may act as antioxidants at low doses but become pro‐oxidant or cytotoxic when present in excess (Rattan 2008; Yang et al. 2024; Vrankova et al. 2026).

One of the most biologically significant outcomes of this study is the biphasic, hormetic effect of several extracts on lifespan. High concentrations of APE‐M and BPE‐M significantly reduced survival, whereas lower concentrations, particularly BPE‐M and BPE‐W at 0.25 μg/mL, extended lifespan. Such non‐monotonic dose–response patterns are characteristic of hormesis, where mild stress activates adaptive cytoprotective pathways that enhance organismal resilience (Rattan 2008; Wan et al. 2024). Similar hormetic longevity effects of plant‐derived bioactive compounds have been reported in Drosophila models (Magwere et al. 2006; Bahadorani et al. 2008; Coşkun and Ayar 2024), and such responses have been associated with conserved stress‐response pathways including Nrf2, FOXO, and mitochondrial adaptive signaling (Tsuji et al. 2024). Whether analogous pathways underlie the lifespan‐extending effects observed in the present study cannot be determined from the current data and warrants investigation in future mechanistic studies.

Recent studies further confirm that dietary plant metabolites can extend fly lifespan through modulation of oxidative stress pathways and metabolic homeostasis (Jaafaru et al. 2024; Qiang et al. 2025; Yuan et al. 2025).

An important observation of this study is that lifespan‐extending extracts did not necessarily increase total antioxidant capacity but instead improved oxidative balance primarily by reducing oxidant burden. For example, BPE‐M markedly decreased TOS and OSI without increasing TAS, suggesting suppression of ROS generation or upregulation of endogenous antioxidant enzymes rather than direct radical scavenging. Such patterns are consistent with a redox‐modulatory mode of action, increasingly recognized as a key mechanism underlying the antiaging effects of plant polyphenols (Sies and Jones 2020; Proshkina et al. 2024). Activation of the Nrf2/ARE pathway by plant‐derived polyphenols has been widely documented in the literature and could potentially be consistent with the observed pattern of reduced TOS and OSI without concomitant increases in TAS (Scuto et al. 2022; Tsuji et al. 2024). However, direct validation of this pathway was beyond the scope of the present study.

With specific reference to *G. elwesii* , the antioxidant‐related effects observed in the present study may be associated with the combined contribution of multiple bioactive constituents. Galantamine, one of the best‐characterized alkaloids reported in this species, is a selective acetylcholinesterase inhibitor that has been associated with neuroprotective and antioxidant‐related effects (He et al. 2015). In addition to alkaloids, *G. elwesii* extracts have been reported to contain phenolic acids and flavonoids with known radical‐scavenging and metal‐chelating properties (Ay et al. 2020). The organ‐dependent biological responses observed in the present study, particularly the comparatively stronger effects of certain belowground extracts, may be consistent with previously reported compositional variation among different Amaryllidaceae plant parts (Berkov et al. 2011; Ay et al. 2020). Collectively, these observations support the possibility that the biological effects of *G. elwesii* extracts involve multiple interacting constituents and pathways. However, the relative contribution of individual compound classes and the precise molecular mechanisms underlying these responses remain unresolved and require validation through targeted mechanistic and bioactivity‐guided studies.

Several findings in the present study warrant critical consideration, as they are not fully consistent with a simple phenolic content–antioxidant activity–bioactivity framework. Most notably, APE‐M exhibited the highest total phenolic and flavonoid content among all extracts yet also produced the greatest developmental toxicity and shortest adult lifespan at high concentrations. Conversely, BPE‐W, which did not rank among the extracts with the highest phenolic content, produced the most pronounced lifespan extension (+15.4% at 0.25 μg/mL). These apparent inconsistencies suggest that total phenolic and flavonoid content alone is insufficient predictors of in vivo biological outcomes in complex plant extracts. It is possible that additional bioactive constituents, extraction‐dependent compositional variability, and concentration‐dependent interactions collectively influenced the observed responses. Furthermore, APE‐W increased TOS and OSI despite moderate phenolic content, indicating a potential pro‐oxidant effect in vivo that further underscores the complexity of crude extract bioactivity. Such discrepancies between in vitro antioxidant capacity and in vivo biological outcomes have been previously reported for plant‐derived extracts and may reflect the multifactorial nature of redox‐associated biological responses under physiological conditions, including concentration‐dependent redox cycling phenomena and ROS‐mediated cellular stress processes (Pisoschi and Pop 2015; Bolaños‐Cardet et al. 2025). Collectively, these observations emphasize that in vitro assays alone cannot reliably predict biological effects and that further studies involving bioactivity‐guided fractionation and compositional characterization would be valuable for clarifying the contribution of individual constituents (Ncube et al. 2008).

Despite its strengths, several limitations should be acknowledged. The use of crude extracts precludes identification of specific active compounds, and chromatographic or spectrometric profiling of individual phenolic and alkaloid constituents was not performed within this study. Previous investigations on *G. elwesii* have reported the presence of biologically relevant alkaloids and phenolic compounds under different extraction and agronomic conditions (Ay et al. 2020, 2023). However, because extract‐specific compositional profiling was not conducted within the current experimental framework, the observed biological effects cannot be directly attributed to individual constituents. Furthermore, TAS, TOS, and OSI represent indirect, integrative markers of systemic oxidative balance and do not provide enzyme‐specific mechanistic information regarding antioxidant defense pathways. Measurement of antioxidant enzymes such as superoxide dismutase (SOD), catalase (CAT), and glutathione peroxidase (GPx), together with additional oxidative damage biomarkers, would provide greater mechanistic resolution in future studies. All lifespan and oxidative stress assays in the present study were conducted exclusively using male Drosophila, as this approach is commonly used to reduce variability associated with female reproductive physiology and related metabolic demands (Magwere et al. 2004). Consequently, the applicability of the present findings to female Drosophila and sex‐dependent physiological responses remains uncertain and should be investigated in future studies. Additionally, oxidative status was assessed using whole‐body homogenates, preventing tissue‐specific analysis of redox effects. Finally, although *D. melanogaster* is a powerful model organism, extrapolation to mammalian systems requires caution (Hales et al. 2015; Ugur et al. 2016).

Overall, the present findings demonstrate that *G. elwesii* extracts exhibit strong organ‐, solvent‐, and dose‐dependent biological effects. Belowground extracts, particularly BPE‐M and BPE‐W, function as effective redox modulators associated with improvements in oxidative balance and lifespan‐related parameters at subtoxic concentrations. These results highlight the importance of hormetic mechanisms and redox signaling pathways in plant‐derived antiaging interventions and underscore the pharmacological potential of *G. elwesii* as a source of bioactive metabolites.

Importantly, this study provides the first systematic in vivo evidence demonstrating that *G. elwesii* extracts exert organ‐dependent hormetic and redox‐modulatory effects that translate into measurable lifespan alterations in a whole‐organism aging model. Unlike previous studies focusing primarily on phytochemical characterization or in vitro antioxidant assays, the present work establishes a direct functional link between phytochemical composition, oxidative balance regulation, and longevity outcomes. The findings further highlight that the biological efficacy of *G. elwesii* is not driven by maximal antioxidant capacity but rather by the ability to modulate systemic redox homeostasis through adaptive stress‐response mechanisms. This integrative approach advances current understanding of Amaryllidaceae bioactivity and identifies belowground extracts of *G. elwesii* as promising candidates for future bioactivity‐guided isolation and development of redox‐targeted antiaging interventions.

## 5 Conclusion

This study provides a comprehensive evaluation of the phytochemical composition, in vitro antioxidant capacity, and in vivo biological effects of *G. elwesii* extracts, revealing pronounced organ‐ and solvent‐dependent variability. Belowground extracts generally exhibited higher phenolic content and stronger antioxidant activities compared to aerial extracts, particularly in radical‐scavenging and metal‐chelation assays. In vivo experiments demonstrated that selected extracts, especially from belowground tissues, can modulate oxidative balance and influence lifespan in *D. melanogaster* in a dose‐dependent manner. Notably, beneficial effects were associated with reduced oxidant burden and improved oxidative status rather than direct antioxidant activity, indicating a primary role in redox regulation and adaptive stress responses. These findings highlight *G. elwesii* as a promising source of bioactive compounds with potential applications in oxidative stress‐related conditions. Future research should focus on bioactivity‐guided fractionation, identification of active constituents, and mechanistic studies to elucidate the molecular pathways underlying their redox‐modulatory and longevity‐associated effects.

## Author Contributions

**Osman Ertan:** conceptualization, investigation. **Arif Ayar:** conceptualization, methodology. **Ebru Batı Ay:** conceptualization, writing – review and editing.

## Funding

This work was supported by Amasya Üniversitesi. Open access funding provided by the Scientific and Technological Research Council of Türkiye (TÜBİTAK).

## Conflicts of Interest

The authors declare no conflicts of interest.

## Data Availability

The data supporting the findings of this study are available from the corresponding author upon reasonable request.
